# How Do Genetic and Environmental Factors Influence Cardiometabolic Risk Factors? Findings from the Isfahan Twins Study

**DOI:** 10.34172/jrhs.2024.139

**Published:** 2024-03-15

**Authors:** Mojgan Gharipour, Minoo Dianatkhah, Shayesteh Jahanfar, Ana Paula dos Santos Rodrigues, Ava Eftekhari, Noushin Mohammadifard, Nizal Sarrafzadegan, Cesar de Oliveira, Erika Aparecida Silveira

**Affiliations:** ^1^Heart Failure Research Center, Cardiovascular Research Institute, Isfahan University of Medical Sciences, Isfahan, Iran; ^2^School of Medicine, Faculty of Health at Deakin University, Melbourne, Australia; ^3^Isfahan Cardiovascular Research Center, Cardiovascular Research Institute, Isfahan University of Medical Sciences, Isfahan, Iran; ^4^Department of Public Health and Community Medicine, Tufts University School of Medicine, Boston, USA; ^5^Graduate Program in Health Sciences, Faculty of Medicine, Federal University of Goiás, Goiânia, Brazil; ^6^Interventional Cardiology Research Center, Cardiovascular Research Institute, Isfahan University of Medical Sciences, Isfahan, Iran; ^7^Faculty of Medicine, School of Population and Public Health, The University of British Columbia, Vancouver, Canada; ^8^Department of Epidemiology and Public Health, Institute of Epidemiology and Health Care, University College London, London, UK

**Keywords:** Genetics, Environment, Cardiometabolic diseases, Risk factors, Obesity, Body mass index

## Abstract

**Background:** Disease-discordant twins are excellent subjects for matched case-control studies as they allow for the control of confounding factors such as age, gender, genetic background, and intrauterine and early environment factors.

**Study Design:** A cross-sectional study.

**Methods:** Past medical history documentation and physical examination were conducted for all participants. Fasting venous blood samples were taken to measure fasting blood glucose (FBG) and lipid levels. The ACE model, a structural equation model, was used to assess heritability.

**Results:** This study included 710 twin pairs (210 monozygotic and 500 dizygotic) ranging in age from 2 to 52 years (mean age: 11.67±10.71 years). The study was conducted using participants from the Isfahan Twin Registry (ITR) in 2017. Results showed that in early childhood (2-6 years), height, weight, and body mass index (BMI) were influenced by shared environmental factors (76%, 75%, and 73%, respectively). In late childhood (7-12 years), hip circumference, waist circumference (WC), and low-density lipoprotein (LDL) cholesterol were found to be highly heritable (90%, 76%, and 64%, respectively). In adolescents, height (94%), neck circumference (85%), LDL-cholesterol (81%), WC (70%), triglycerides (69%), weight (68%), and BMI (65%) were all found to be highly or moderately heritable. In adult twins, arm circumference (97%), weight (86%), BMI (82%), and neck circumference (81%) were highly heritable.

**Conclusion:** This study demonstrates that both genetic and environmental factors play a role in influencing individuals at different stages of their lives. Notably, while certain traits such as obesity have a high heritability during childhood, their heritability tends to decrease as individuals transition into adulthood.

## Background

 Cardiovascular diseases (CVDs) are the leading cause of global mortality and a significant contributor to disability. In Iran, they account for 46% of all deaths and about 22% of the burden of disease.^1^ The INTERHEART study investigated various traditional and emerging risk factors in 27 000 individuals with CVD from 52 countries and identified nine risk factors that accounted for approximately 90% of the population-attributable risk of CVD.^2^

 Cardiometabolic risk factors refer to factors that increase the likelihood of experiencing vascular events or developing diabetes.^3^ Previous genetic studies have identified a list of candidate genes to consider in gene-lifestyle research concerning CVD and investigated whether they can influence an individual’s response to lifestyle interventions. While we recognize that the genetic architecture associated with cardiometabolic risk factors may encompass common and rare genetic variants, copy number variation, and epigenetic mechanisms, most research has focused on common single nucleotide polymorphisms.^4^ The heritability of cardiometabolic risk factors has been estimated to range from 20% to 60%, with the remaining 40% attributed to environmental factors.^5^ This wide range could be attributed to differences in study design, setting, and population.

 Twin studies comparing individual traits of monozygotic (MZ) and dizygotic (DZ) twins can provide insights into the respective influence of environment and genetics. MZ twins share not only all their genes but also their childhood and early environment, while DZ twins share, on average, only 50% of their genes.^4^ Consequently, any dissimilarity between MZ twins is assumed to be due to non-shared environmental differences, whereas dissimilarity between DZ twins is attributed to both genetic and non-shared environmental factors. Therefore, if MZ twins exhibit greater similarity in relation to cardiometabolic risk factors compared to DZ twins, it can be concluded that there is a genetic contribution to the trait.^6^ Since the level of genetic and environmental influences on cardiometabolic risk factors can vary in different populations, this study aims to evaluate cardiometabolic risk factors in Iranian MZ and DZ twin pairs without known CVDs in order to determine the genetic and environmental influences on these factors.

## Materials and Methods

###  Study population

 The Isfahan Twin Registry (ITR) is a population-based registry of twins living in Isfahan province, Iran, which was established in 2017. Participants were categorized into six groups: infants (1-24 months), early childhood (2-6 years), late childhood (7-12 years), adolescents (13-18 years), adults (19 years and older), and parents of children under six years. The enrolment process and methodology of the ITR have been previously published.^7^ The study was approved by the Research Ethics Committee of Isfahan University of Medical Sciences (IR.mui.med.rec.1399.169), and all participants provided informed consent before participating in the study.

###  Data collection

 Sociodemographic data, including age, sex, and family socioeconomic status, were collected. The following tools were used to assess various aspects: a 106-item Food Frequency Questionnaire (FFQ) to evaluate dietary habits,^8^ the International Physical Activity Questionnaire short form (IPAQ-SF) to assess physical activity,^9^ and the Iranian-validated version of the Hospital Anxiety and Depression Scale (HADS) to evaluate psychological status.^10^ Data on participants’ disease history and pharmacological treatment were obtained from their medical records, including hospitalization records, surgeries, and para-clinical tests. Smoking status and nicotine dependence were also recorded.

###  Physical measurements 

 Weight was measured to the nearest 0.5 kg using a scale with minimal clothing. Height was measured to the nearest 0.5 cm using a non-elastic meter while participants stood barefoot with their shoulders in a neutral position. Waist circumference (WC) was measured at a level midway between the lower rib margin and the iliac crest with a tape measure, and hip circumference was measured at the point of maximum circumference over the buttocks using a non-elastic meter.^11^ Trained nurses measured blood pressure using the standard protocol defined by the Joint National Committee (JNC-8). Systolic blood pressure (SBP) and diastolic blood pressure (DBP) were recorded as the average of the two measures of the first and fifth Korotkoff phases using Omron digital blood pressure monitor.^12^

###  Biochemical Measurements

 Fasting blood glucose (FBG), total cholesterol (T-Chol), triglyceride (TG), high-density lipoprotein (HDL) cholesterol, and low-density lipoprotein (LDL) cholesterol were measured using standard enzymatic kits (Pars Azmoon, Iran) and an analyzer (Hitachi 902, Japan) in the central laboratory of Isfahan Cardiovascular Research Institute, a World Health Organization (WHO) collaborating center. LDL cholesterol level was measured directly.

###  Measurement of cardiometabolic risk factors

 Cardiometabolic risk factors included blood pressure, blood lipid profile (total cholesterol, LDL cholesterol, HDL cholesterol, and triglycerides), blood glucose levels, and anthropometric measurements (body mass index [BMI], WC, etc).

###  Statistical analysis

 Zygosity was determined based on the validated questionnaire developed by Song et al using the decision tree method.^13^ Descriptive statistical analysis was used to present the characteristics of twin pairs. Bivariate correlations between the same age- and gender-corrected parameters were separately analyzed in MZ and DZ pairs. A *P *value < 0.05 was considered indicative of statistical significance. A genetic effect was assumed when coefficients of correlations (r values) were high in MZ and not in DZ pairs. On the other hand, if “r” values were lower or close to each other when MZ and DZ pairs were compared, an environmental effect was assumed. Fisher’s z-test was used to find differences between correlations in MZ and DZ.^14^ For assessing heritability, the ACE model, as a structural equation model, was used. In this model, three latent variables, additive genetic effects (A), common (or shared) environmental factors (C), and unshared (or unique) environmental factors (E), drive the variance in the phenotype for each twin.^13^ Classical twin models can also estimate, for a given phenotype, the proportion of variance due to genetics (the sequence of DNA itself), shared environment (all exposures that twins share), and non-shared environment (exposures and stochastic effects that differ within pairs). They use statistical techniques in which phenotypic variance is partitioned into additive genetic effects (abbreviated A), common or shared environmental effects (C), and non-shared or unique environmental effects (E).^15^ These three types of variance are usually expressed as ratios that add up to one. “Additive” implies that the number of genetic variants correlates with the severity of the phenotype linearly. Additive factors, along with other genetic effects such as dominance, are used in estimating heritability. Heritability is a proportion rather than an absolute number, while genotype is primarily fixed at conception. Therefore, the estimate of heritability is inversely proportional to the total variation in phenotype that can be increased by geographic and age-related factors. For example, height is less heritable in developing countries with higher environmental adversity than in developed countries with less adversity.^16^ At the same time, the heritability of BMI increases from birth to childhood and then declines in adulthood.^15^ “A” is perfectly (1.0) correlated across MZ twins and 0.5 correlated across DZ twins. “C” represents a perfectly correlated within twin pairs independently of zygosity. “E” is uncorrelated across co-twins.^17^ Estimates and their 95% confidence intervals (CIs) were calculated. Heritability analyses were performed using the package METs in R version 4.3.2 (2023-10-31 ucrt).^13^

## Results

 The final analytical sample consisted of 710 twins, with a mean age of 11.67 ± 10.71 years. Table 1 presents the number of participants by gender and zygosity in each age group. Of the total participants, 210 were MZ twins and 500 were DZ twins. Table 2 displays the bivariate correlations (r values and their 95% CIs) between age and gender-corrected parameters measured in MZ and DZ twin pairs. *P* values between MZ and DZ were reported for all variables. A high coefficient of correlation in MZ pairs and not in DZ pairs suggests a genetic effect, whereas lower or similar coefficients indicate an environmental effect.

**Table 1:** Distribution of twins based on the zygosity and gender

**Age group (year**)	**Monozygotic, n=210**		**Total**	**Dizygotic, n=500**			**Total**
	**Girl-girl**	**Boy-boy**		**Girl-girl**	**Boy-boy**	**Girl-boy**	
Early childhood (2-6)	44	28	72	72	62	80	214
Late childhood (7-12)	26	32	58	58	34	68	160
Adolescence (13-18)	14	4	18	26	8	10	44
Adulthood ( > 18)	36	26	62	40	24	18	82

**Table 2:** Bivariate correlation (r values and 95% CIs) in age- and gender-corrected parameters measured in monozygotic (MZ) and dizygotic (DZ) twin pairs based on different age groups

**Variables**	**Group**	**r values (95% CI) based on age groups (yr)**			
		**2-6**	**7-12**	**13-18**	**>18**
FBG	MZ	0.82 (0.64, 0.91)	0.69(0.48,0.82)	0.79 (0.50, 0.92)	0.32 (0.05, 0.54)
	DZ	0.41 (0.34, 0.47)	0.46(0.28,0.61)	0.62 (0.28, 0.82)	0.32 (0.05, 0.54)
	*P *value	0.001	0.122	0.463	0.999
T-Chol	MZ	0.73 (0.56, 0.84)	0.81 (0.67, 0.90)	0.63 (0.37, 0.80)	0.47 (0.23, 0.66)
	DZ	0.61 (0.48, 0.72)	0.54 (0.38, 0.67)	0.63 (0.37, 0.80)	0.47 (0.23, 0.66)
	*P *value	0.274	0.021	0.999	0.999
LDL	MZ	0.61 (0.48, 0.70)	0.82 (0.69, 0.90)	0.81 (0.21, 0.97)	0.66 (0.39, 0.83)
	DZ	0.61 (0.48, 0.70)	0.50 (0.33, 0.64)	0.41 (0.24, 0.55)	0.58 (0.31, 0.76)
	*P *value	0.999	0.007	0.144	0.607
HDL	MZ	0.74 (0.65, 0.81)	0.83 (0.71, 0.90)	0.56 (0.06, 0.83)	0.80 (0.68, 0.88)
	DZ	0.74 (0.65, 0.81)	0.52 (0.35, 0.65)	0.54 (0.18, 0.77)	0.80 (0.68, 0.88)
	*P *value	0.999	0.007	0.954	0.999
TG	MZ	0.52 (0.38, 0.63)	0.57 (0.25, 0.78)	0.69 (0.28, 0.88)	0.80 (0.67, 0.88)
	DZ	0.52 (0.38, 0.63)	0.34 (0.16, 0.51)	0.34 (0.19, 0.48)	0.80 (0.67, 0.88)
	*P *value	0.999	0.194	0.295	0.999
Weight	MZ	0.95 (0.91, 0.97)	0.88 (0.79, 0.93)	0.97 (0.93, 0.99)	0.90 (0.82, 0.94)
	DZ	0.85 (0.79, 0.89)	0.59 (0.44, 0.71)	0.63 (0.31, 0.82)	0.47 (0.21, 0.67)
	*P *value	0.003	0.002	0.003	0.001
Height	MZ	0.97 (0.95, 0.98)	0.90 (0.83, 0.94)	0.94 (0.83, 0.98)	0.77 (0.62, 0.87)
	DZ	0.87 (0.81, 0.90)	0.70 (0.58, 0.79)	0.47 (0.44, 0.50)	0.74 (0.58, 0.85)
	*P *value	0.001	0.008	0.009	0.786
BMI	MZ	0.96 (0.94, 0.98)	0.88 (0.79, 0.93)	0.97 (0.91, 0.99)	0.88 (0.78, 0.93)
	DZ	0.85 (0.79, 0.89)	0.65 (0.52, 0.76)	0.64 (0.35, 0.82)	0.47 (0.20, 0.67)
	*P *value	0.001	0.008	0.004	0.001
WC	MZ	0.78 (0.63, 0.87)	0.87 (0.78, 0.93)	0.89 (0.70, 0.96)	0.70 (0.57, 0.80)
	DZ	0.46 (0.32, 0.59)	0.49 (0.32, 0.64)	0.54 (0.20, 0.76)	0.70 (0.57, 0.80)
	*P *value	0.006	0.001	0.080	0.999
HP	MZ	0.88 (0.79, 0.93)	0.90 (0.83, 0.95)	0.96 (0.88, 0.98)	0.19 (0.04, 0.40)
	DZ	0.61 (0.49, 0.71)	0.45 (0.42, 0.48)	0.65 (0.36, 0.82)	0.19 (0.04, 0.40)
	*P *value	0.001	0.001	0.012	0.999
NC	MZ	0.50 (0.37, 0.61)	0.56 (0.42, 0.68)	0.85 (0.57, 0.96)	0.83 (0.70, 0.91)
	DZ	0.50 (0.37, 0.61)	0.56 (0.42, 0.68)	0.43 (0.34, 0.51)	0.43 (0.20, 0.61)
	*P *value	0.999	0.999	0.090	0.003
AC	MZ	0.74 (0.65, 0.80)	0.84 (0.72, 0.91)	0.82 (0.53, 0.94)	0.35 (0.03, 0.65)
	DZ	0.74 (0.65, 0.80)	0.59 (0.43, 0.71)	0.52 (0.18, 0.75)	0.18 (0.00, 0.34)
	*P *value	0.999	0.020	0.215	0.460
SBP	MZ	0.80 (0.67, 0.88)	0.70 (0.59, 0.79)	0.47 (0.15, 0.70)	0.51 (0.32, 0.67)
	DZ	0.50 (0.35, 0.63)	0.70 (0.59, 0.79)	0.47 (0.15, 0.70)	0.51 (0.32, 0.67)
	*P *value	0.006	0.999	0.999	0.999
DBP	MZ	0.49 (0.24, 0.68)	0.54 (0.39, 0.66)	0.14 (0.21, 0.47)	0.45 (0.17, 0.66)
	DZ	0.45 (0.28, 0.59)	0.54 (0.39, 0.66)	0.14 (0.21, 0.47)	0.44 (0.13, 0.67)
	*P *value	0.801	0.999	0.999	0.961

FBG, fasting blood glucose; T-Chol, total cholesterol; LDL, low density lipoprotein.; HDL, high density lipoprotein; TG, triglyceride, BMI, body mass index; WC, waist circumference; HC, hip circumference; NC, neck circumference; AC, arm circumference; SBP, systolic blood pressure; DBP, diastolic blood pressure.

 When assessing the bivariate correlation between age- and gender-corrected parameters measured separately in MZ and DZ twin pairs, we found that the coefficients of correlation (r values) for FBG were higher in MZ twins than in DZ twins only during early childhood (*P* < 0.001). No significant difference was observed in other age groups (*P* > 0.05). For the second age group, lipid profiles, apart from TG, showed higher r-values in MZ twins. Weight and BMI were influenced more by genetics than by the environment in all age groups. On the other hand, height, WC, and hip circumference were primarily influenced by genetics until 18 years old. However, in adults, only neck circumference showed a clear genetic influence. Arm circumference was influenced by genetics around the time of puberty. There was no evidence of genetic influence on DBP, and SBP showed an environmental effect in all age groups except for the first one (Table 2).

**Figure 1:**
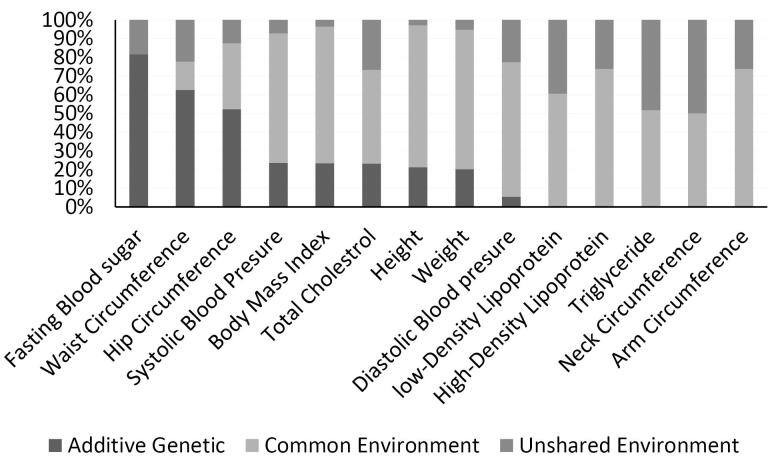
Percentage of additive genetic (A), common (C) and unique environmental (E) influences on cardiometabolic risk factors (univariate structural equation model [ACE] analysis in 286 twin pairs in early childhood)

**Figure 2:**
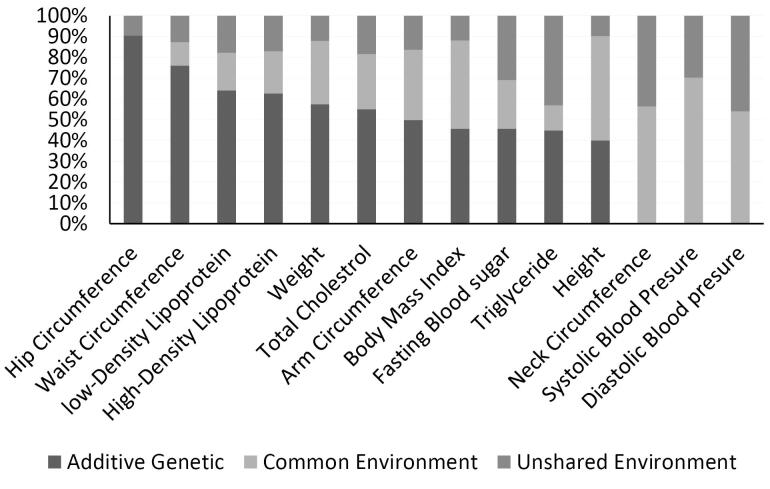
Percentage of additive genetic (A), common (C) and unique environmental (E) influences on cardiometabolic risk factors (univariate structural equation model [ACE] analysis in 218 twin pairs in late childhood)

**Figure 3:**
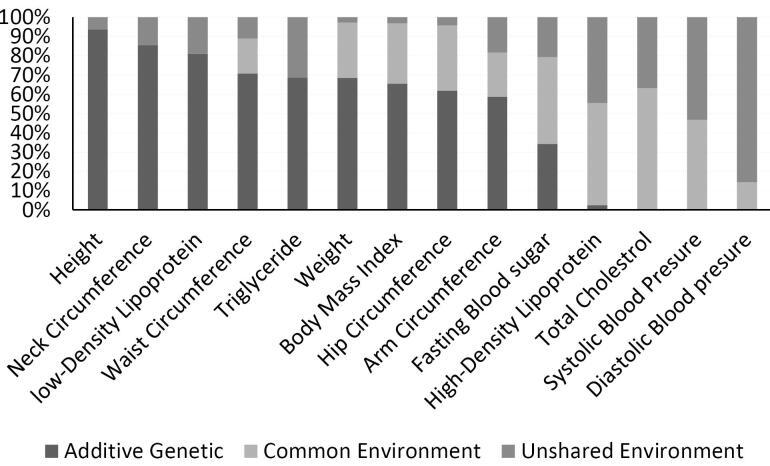
Percentage of additive genetic (A), common (C) and unique environmental (E) influences on cardiometabolic risk factors (univariate structural equation model [ACE] analysis in 62 adolescent twin pairs)

 The ACE model demonstrated that the following risk factors were highly or moderately heritable in early childhood: FBG [0.81(0.68, 0.94)], WC [0.62(0.28, 0.96)], and hip circumference [0.52(0.28, 0.75)]. Conversely, shared environment influences were found on height [0.75(0.67, 0.84)], weight [0.74(0.64, 0.84)], body mass index [0.73(0.63, 0.82)], SBP [0.69(0.56, 0.81)], HDL [0.73(0.65, 0.81)], and triglycerides [0.51(0.38, 0.64)] (Figure 1). In late childhood (218 twin pairs), hip circumference [0.90(0.84, 0.95)], WC [0.75(0.43, 1.08)], and LDL [0.63(0.29, 0.98)] were highly influenced by genetic factors. Meanwhile, SBP [0.70(0.60,0.79)] and DBP [0.53(0.40, 0.67)] were influenced by shared and common environmental factors (Figure 2). In adolescents, height [0.93(0.871, 1.00)], neck circumference [0.85(0.68, 1.02)], LDL [0.81(0.49, 1.12)], WC [0.70(0.13,1.27)], BMI [0.81(0.34, 1.28)], and neck circumference [0.81(0.37, 1.24)] were highly heritable. However, HDL [0.79(0.69, 0.89)] and TG [0.8(0.69, 0.9)] had the highest proportion of total phenotypic variance due to shared and unique environmental influences in this age group (Figure 3).

**Figure 4:**
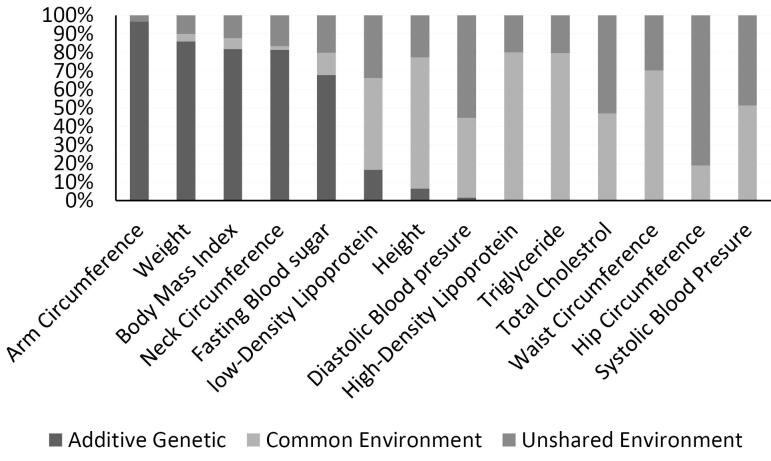
Percentage of additive genetic (A), common (C) and unique environmental (E) influences on cardiometabolic risk factors (univariate structural equation model [ACE] analysis in 144 adult twin pairs)

 In adults (144 twin pairs), anthropometric measurements including arm circumference [0.96(0.94, 0.98)], weight [0.85(0.399,1.31)], body mass index [0.81(0.34,1.28)], and neck circumference [0.81(0.37, 1.24)] were more influenced by genetic factors. However, in this age group, HDL [0.79(0.69, 0.89)] and triglycerides [0.8(0.69,0.9)] had the highest proportion of total phenotypic variance due to shared and unique environmental influences (Figure 4).

## Discussion

 To the best of our knowledge, this is the first study to assess the influence of genetic and environmental factors on cardiometabolic risk factors in Iranian twins. Our main findings indicate that the heritability of cardiometabolic traits differs at different stages of life. The effect of heredity on some cardiometabolic risk factors at early age is greater than 50%, but the effect of common and unshared environmental factors gradually increases throughout life. Previous population-based genome-wide association studies have highlighted the role of genetic determinants in many complex diseases, such as diabetes and CVD. At the same time, numerous meta-analyses and metagenomic analyses have failed to demonstrate a significant relationship between genes and complex diseases ^18^. These findings encourage scientists to focus on the role of epigenetic modifications in clarifying the interaction between genetic and environmental factors.

 FBG in the first two years of life is more than 80% affected by heredity, but in late childhood and adolescence, the effect of heredity decreases by 50% and 30%, respectively. Furthermore, the influence of heritability on FBG steadily decreases from early childhood to adolescence (80% to 30%), and in adulthood, FBG is almost 60% influenced by genetic factors. In support of our findings, Wang et al^19^ established a significant interaction between the genetic variation of FBG and nutritional intervention on changes in glycaemic traits in adults. However, findings from a study conducted by Simonis-Bik et al did not corroborate other twin studies. They showed that genetic factors do not influence glucose metabolism indicators such as glycated haemoglobin (HbA1c) and FBG.^20^

 Obesity is another cardiometabolic risk factor that increases the risk of many chronic diseases, such as diabetes, CVD, and cancers.^21^ We found that anthropometric parameters, including weight, waist, and BMI, are strongly influenced by heritability, which is in line with previous evidence.^22-24^ The ACE analysis demonstrated that anthropometric indices are mostly heritable in the first two years of life. Nevertheless, with increasing age, the effect of common and unshared environmental factors increases gradually for this trait. In adolescence, anthropometric measurements such as weight, BMI, and neck circumference are more influenced by environmental factors than genetics. In line with our findings, a meta-analysis on twin studies by Elks et al reported that BMI heritability estimates could range from 0.47 to 0.90.^17^ However, a meta-regression showed that the BMI heritability estimate was 0.07 higher in children than in adults (*P* = 0.001). Interestingly, in adulthood, arm circumference is significantly influenced by genetic factors.

 Adolescence is a transitional phase from childhood to adulthood with apparent changes in behavior and lifestyle. Previous research has highlighted the impact of changes in the level of sex hormones after puberty and their impact on obesity indices.^25^ Remarkably, in adulthood, there is substantial heritability of anthropometric parameters (except for WHR and WC), which is in line with previous investigations.^26^ Based on these results, we suppose that the change is remarkably due to sexual dimorphism. In adulthood, sexual hormones could affect the shape of the body.

 One of the earliest twin anthropometric studies conducted by Chatterjee et al (1999) demonstrated that differences between MZ and DZ in anthropometric measurements could be due to differences in gender, age, nutritional status, socioeconomic and nutritional factors, and genetic factors.^27^ One of the ongoing debates in life-course epidemiology is whether an adverse prenatal environment, indicated by birth weight, increases the risk of cardiometabolic disease in the future, which has been investigated by scientists in the UK Biobank.^27^ They conducted a discordant twin study to control for shared genetic and environmental factors. The results of their study suggested that there is no strong evidence supporting a causal relationship between birth weight and most cardiometabolic risk factors later in life. However, nominal associations were found with C-reactive protein and insulin-like growth factor 1, although these associations were not replicated in dizygotic twin pairs. Therefore, this study does not provide support for the hypothesis that adverse prenatal environments increase the risk of cardiometabolic disease in later life. Another study by Hong et al sought to find evidence regarding the relative roles of genes and the environment underlying obesity and cardiometabolic disease traits, as well as the correlations between them and the way they change with age. The results of their study revealed a high heritability of BMI (72%), with cardiometabolic traits ranging from 30% (HbA1c) to 69% (HDL-C). As age increased, the heritability of all phenotypes showed varying degrees of decline, with BMI, SBP, and DBP displaying significant monotonous declining trends. Bivariate structural equation model (SEM) s indicated that BMI was correlated with all cardiometabolic traits, with genetic correlations ranging from 0.14 (BMI and LDL-C) to 0.39 (BMI and DBP) and environmental correlations ranging from 0.13 (BMI and TC/LDL-C) to 0.31 (BMI and TG). The genetic contributions underlying the correlations between BMI and SBP, DBP, TC, TG, and HDL-C showed a progressive decrease as age groups increased. In contrast, environmental correlations exhibited a significant increasing trend for HbA1c, SBP, and DBP. Their findings suggest that both genetic and environmental factors have significant effects on BMI and all cardiometabolic traits. However, as age groups increase, genetic influences show varying degrees of decrement for BMI and most cardiometabolic traits, indicating the increasing importance of environmental factors. Genetic factors consistently play a larger role than environmental factors in the phenotypic correlations between BMI and cardiometabolic traits. Nevertheless, the relative magnitudes of genetic and environmental factors may change over time.^28^

 The highest heritability rate in lipids was related to LDL, which was 80% in adolescence. Common and unshared environments influence lipids such as TG, HDL, and T-chol. This is in line with previous twin studies.^29^ In our participants, SBP was more heritable in early childhood, and with increasing age, the effect of the common and unshared environment on this trait increased. The influence of an unshared environment on the mentioned traits varied from 3% to 45%.^30^

 Our study has some limitations that should be acknowledged. It was difficult to find and convince the twins and their families to participate. The sample size was small in each age group; however, they were comparable to other twin studies. In addition, our participants were healthy and mostly young twins, so the obtained results are limited to adolescence and early adulthood. The strengths of our study include the use of a simple random sampling method to avoid selection bias, the stratification of the whole sample by age group, and the investigation of all subjects by the same investigators in one research institute. Furthermore, all laboratory parameters were centrally measured in the same hospital immediately after taking the blood sample.

**Highlights**

- The study highlights the dynamic nature of genetic and environmental influences on individuals throughout different life stages.
- Obesity displayed high heritability in childhood but it showed a decrease in heritability as individuals transitioned into adulthood.
- Genetic factors strongly impact FBS in early childhood and adulthood but not in late childhood and adolescence.

## Conclusion

 In our study, we observed that various factors, both genetic and environmental, have an impact on individuals at different stages of their lives. Particularly, we found that certain traits, such as obesity, have a high heritability during childhood but their heritability tends to decline as one progresses into adulthood.

## Acknowledgments

 This registry is funded by Isfahan University of Medical Sciences and Isfahan Cardiovascular Research Center (Number 97101). The authors would like to thank Isfahan Welfare Organization and Persian Twins Society (a non-governmental organization). We also acknowledge the laboratory staff of the Cardiovascular Research Institute. Additionally, we appreciate the volunteers who participated in the study and generously donated their time to the registry.

## Authors’ Contribution

**Conceptualization:** Nizal Sarrafzadegan, Mojgan Gharipour.

**Data curation:** Minoo Dianatkhah.

**Formal analysis:** Minoo Dianatkhah.

**Funding acquisition:** Mojgan Gharipour.

**Investigation:** Nizal Sarrafzadegan, Mojgan Gharipour, Shayesteh Jahanfar, Noushin Mohammadifard.

**Methodology:** Mojgan Gharipour.

**Project administration:** Mojgan Gharipour.

**Resources:** Ava Eftekhari.

**Software:** Minoo Dianatkhah.

**Supervision:** Nizal Sarrafzadegan

**Validation:** Noushin Mohammadifard, Ava Eftekhari

**Visualization:** Shayesteh Jahanfar, Ana Paula dos Santos Rodrigues, Noushin Mohammadifard, Nizal Sarrafzadegan, Cesar de Oliveira, Erika Aparecida Silveira.

**Writing–original draft:** Mojgan Gharipour, Minoo Dianatkhah, Cesar de Oliveira, Erika Aparecida Silveira, Nizal Sarrafzadegan, Shayesteh Jahanfar.

**Writing–review & editing:** Mojgan Gharipour, Minoo Dianatkhah.

## Competing Interests

 None.

## Ethical Approval

 The Research Ethics Committee of Isfahan University of Medical Sciences approved the Isfahan Twin Registry (IR.mui.med.rec.1399.169). All participants provided informed consent to participate in the clinical examination. After obtaining informed written consent, a medical interview and physical examination were conducted.

## Funding

 This study was financially supported by Isfahan Cardiovascular Research Institute, Isfahan University of Medical Sciences, Isfahan, Iran.
